# Diagnostic yield and accuracy of paediatric image-guided fine needle aspiration biopsy of deep organ tumours

**DOI:** 10.4102/sajr.v26i1.2485

**Published:** 2022-09-28

**Authors:** Tichayedza Bondera, Pawel Schubert, Anel van Zyl, Richard Pitcher, Asif Bagadia

**Affiliations:** 1 Division of Radiodiagnosis, Department of Medical Imaging and Clinical Oncology, Faculty of Medicine and Health Sciences, Stellenbosch University, Cape Town, South Africa; 2 Division of Anatomical Pathology, Faculty of Medicine and Health Sciences, Tygerberg Hospital, Stellenbosch University, Cape Town, South Africa; 3 Department of Paediatrics and Child Health, Faculty of Medicine and Health Sciences, Stellenbosch University, Cape Town, South Africa

**Keywords:** paediatric, image-guided fine needle aspiration and biopsy (FNAB), rapid on-site evaluation (ROSE), diagnostic yield, diagnostic accuracy, deep organ tumours

## Abstract

**Background:**

Paediatric tumour cytological diagnosis by image-guided fine needle aspiration biopsy (FNAB) with rapid on-site evaluation (ROSE) has not gained wide acceptance despite increasing publications advocating the procedure.

**Objective:**

The primary aim was an audit of the diagnostic yield and accuracy of paediatric image-guided FNAB with ROSE at a single institution. Evaluation of safety was a secondary aim.

**Method:**

Details of consecutive cases of paediatric image-guided FNAB with ROSE for suspected non-benign deep-seated lesions performed from 01 January 2014 to 30 April 2020 were retrieved from the institutional radiology and laboratory databases. Diagnostic yield and accuracy were evaluated using clinico-pathological-radiological correlation and/or subsequent histological specimen diagnosis correlation. Complications and the frequency of key radiological features potentially affecting yield and accuracy were described.

**Results:**

Of 65 cases retrieved, cytology showed malignancy in 52, benign features in five and one indeterminate diagnosis; seven samples were insufficient for cytological assessment. Of the 65 cases, 58 had subsequent formal histological diagnosis. The overall diagnostic yield was 98.5%, with 94.5% sensitivity, 100.0% specificity, 100.0% positive predictive value, 75.0% negative predictive value and 95.3% diagnostic accuracy. All cases (*n* = 26) demonstrating restricted diffusion on MRI yielded adequate samples and cyto-histopathological correlation.

**Conclusion:**

Paediatric image-guided FNAB with ROSE has a relatively high diagnostic yield, sensitivity, specificity, positive predictive value and accuracy in the diagnosis of deep-seated tumours. The relatively low negative predictive value may reflect insufficient samples obtained from cystic and/or benign lesions. Sampling from areas of restricted MRI diffusion may enhance diagnostic yield.

## Introduction

Image-guided percutaneous biopsy is frequently performed in interventional radiology departments by way of core needle biopsy (CNB) or fine needle aspiration biopsy (FNAB).^1^ These techniques utilise available imaging technology to improve the safety of needle insertion into deep visceral organs and tissues for biopsies that would otherwise require open or laparoscopic surgery. Of note, some patients with advanced disease or certain comorbidities may be unfit for surgery. Furthermore, theatre time is limited in resource-constrained environments.

Various image-guidance modalities are available, including ultrasonography (US), CT, MRI, fluoroscopy and newer commercially available navigation systems.^1^ The preferred modality is dependent on the interventional radiologist’s experience, available imaging equipment and the site of the lesion.

The success of these minimally invasive procedures rests on their safety, high diagnostic yield and rapid recovery time.^1^ They facilitate pathological tissue diagnosis and assessment of tumour biomarkers with a lower risk of severe complications than open surgical biopsies (< 1% versus 2% – 10%).^2^ In addition, as imaging techniques have advanced, the ability to detect and target smaller lesions has improved, allowing earlier diagnosis and treatment.^3^

Adult data suggest that image-guided biopsies are faster and less invasive, and thus safer and cheaper than open surgical incisional biopsy.^4^ Although open biopsy has historically provided better diagnostic yield, FNAB and CNB have generally become the first-line procedures without sacrificing diagnostic outcome.^4^

Although CNB yields more tissue for evaluation, it is generally performed with a larger-bore needle (14- to 20-gauge; outer diameter 2.1 mm – 0.91 mm) whereas FNAB is typically defined by the use of a ≥ 22 gauge needle (outer diameter ≤ 0.72 mm).^5^ Larger needles have the potential for greater tissue disruption and hence increased risk of tumour tract seeding, haemorrhage, pain and need for more formal anaesthesia.^6^ Furthermore, compared to FNAB, there is less provision for on-site evaluation of CNB sample adequacy and the cost is higher.^6^

Fine needle aspiration biopsy advantages include rapidity of diagnosis, cost-effectiveness, multiple sampling (including primary and metastatic sites) and the inclusion of small lesions such as lymph nodes. The capacity for numerous ancillary investigations, namely immunocytochemical stains and molecular investigations has enabled FNAB to achieve high sensitivity and specificity.^7^ Rapid on-site evaluation (ROSE) is a cytopathologic diagnostic adequacy assessment of individual biopsy passes performed during a biopsy procedure in order to optimise the procedure itself and inform subsequent patient management.^8^ It improves diagnostic yield while minimising the risk of multiple needle passes.^1^ Furthermore, therapeutic aspiration is also possible in cases with cysts, which is not possible with CNB.^6^

While certain studies have documented higher FNAB sensitivity compared to CNB,^9,10^ others have shown the converse.^11,12^ These discrepant reports highlight the challenge of accurately determining relative performance due to the interplay of multiple objective and subjective parameters.^6,7^ These include operator factors (radiologist and cytopathologist experience), the availability of cytopathology ROSE, lesional features (palpability, size, overt malignancy) and imaging equipment performance.^6,7^

Primary paediatric tumour cytological diagnosis by image-guided FNAB with ROSE has not gained wide acceptance despite increasing publications in support of the procedure.^13^ This procedure has the potential to shorten the time to definitive diagnosis thereby facilitating an earlier start to treatment, particularly in institutions where paediatric surgery theatre time is limited.

In this study at a tertiary South African academic centre, we retrospectively audited consecutive paediatric image-guided FNAB with ROSE to evaluate the procedure’s diagnostic yield and accuracy. These parameters were assessed by clinico-pathological-radiological correlation with treatment response and follow-up where needed and/or subsequent histological specimen diagnosis.

## Research methods and design

This was a five-year, retrospective, descriptive, non-experimental, cross-sectional study conducted from 01 January 2014 through 30 April 2020 at Tygerberg Hospital, Cape Town, South Africa. All consecutive image-guided FNAB procedures with ROSE for deep organ tumours in children 0–17 years were included. Cases without histological confirmation or adequate clinico-pathological-radiological correlation were excluded. The anonymised data collection form included demographic and clinical data, the anatomical site, radiological characteristics of the lesion, procedural complications, FNAB and ancillary pathological test results and the histopathological diagnosis. Clinical information was obtained from patient records, radiology images and results were obtained from the Picture Archiving and Communication System (PACS) electronic radiology platform and pathology laboratory results from the National Health Laboratory Services electronic platform.

The majority of the paediatric FNAB procedures were synchronised with the imaging studies. Most lesions were located in the abdomen, and thus typically required staging MRI abdomen and CT chest studies. At our institution these studies are performed sequentially, most commonly under general anaesthesia or conscious sedation. This provides an opportunity for synchronised sampling while the patient is still on the CT machine table, that is, immediately after the staging CT chest study, and takes advantage of the same anaesthesia. In our experience, this results in increased operational efficacy, without sacrificing patient safety standards. Alternatively, for the remainder of cases or where patients already had the required imaging studies done at peripheral centres, the patients were booked on a dedicated elective paediatric interventional radiology list done once a week under general anaesthesia. Post-procedure, the patients were monitored overnight for complications in the paediatric oncology unit and discharged the following day if continued admission was not required.

All FNAB cases in this cohort were performed with ultrasound guidance utilising a 22-gauge needle. Biopsies were conducted by the interventional radiology team, with ROSE evaluation by the attending cytopathologist. Histology where available, as well as relevant clinical and laboratory correlation were used as the control for the measurement of the study question. Cases with no clinical, laboratory and histology correlation were excluded.

### Statistical analysis

For statistical computation purposes, both the cytological and surgical diagnoses were categorised into four groups: benign, atypical/indeterminate, malignant, and insufficient for diagnosis (Table 1). ‘Atypical’ and ‘suspicious for malignancy’ were considered and categorised as ‘positive for tumour’ for statistical computation purposes. Each of these lesions was assigned to a specific diagnostic category (True positive, True negative, False positive, False negative or excluded).

**TABLE 1:** Summarised results and diagnostic categories.

Cytological diagnosis	Histological, laboratory or clinically confirmed diagnosis	Number of cases	Category
Benign lesions (*n* = 5)	Not available	0	Excluded
	Non-neoplastic	4†	True negative
	Benign lesion	1‡	True negative
	Atypical (indeterminate)	0	Excluded
	Malignant neoplasm	0	False negative
Atypical/suspicious/indeterminate (*n* = 1)	Not available	0	Excluded
	Non-neoplastic	0	False positive
	Benign lesion	0	False positive
	Atypical (indeterminate)	0	Excluded
	Malignant neoplasm	1§	True positive
Malignant neoplasm (*n* = 52)	Not available	1¶	Excluded
	Non-neoplastic	0	False positive
	Benign lesion	0	False positive
	Atypical (indeterminate)	0	Excluded
	Malignant neoplasm	51	True positive
Insufficient for diagnosis (*n* = 7)	Not available	0	Excluded
	Non-neoplastic	1††	True negative
	Benign lesion	3‡‡	True negative
	Atypical (indeterminate)	0	Excluded
	Malignant neoplasm	3§§	False negative

**Total number of cases**		**65**	**-**

† , Abscess, chronic inflammation, acute on chronic inflammation, mild inflammation of the salivary gland;

‡ , Normal liver;

§ , Cystic nephroblastoma;

¶ , Nephroblastoma;

†† , Retro areolar breast cyst;

‡‡ , Arteriovenous malformation, benign fibro-connective tissue, benign mature teratoma;

§§ , Cystic nephroblastoma, desmoid fibromatosis, primary medullary lymphoma.

The sensitivity, specificity, diagnostic yield, accuracy, negative and positive predictive values were calculated as a proportion of the total cases. Basic descriptive statistics were used to describe demographic data, tumour type, site and size.

### Ethical considerations

The study was approved by the Stellenbosch University Health Research Ethics Committee (HREC1-2021-13195). A waiver of informed consent was obtained as the risk to participants for participating in this retrospective study was minimal. Data was anonymised by the allocation of a unique study number.

## Results

Sixty-five cases met the inclusion criteria, with only one case excluded from the calculation of the study question due to patient demise prior to both histological confirmation and adequate clinico-pathological-radiological correlation. In this study cohort (male:female = 1.0:2.5), the mean age was 47.5 months, median age 34 months, range of five days to 17 years and interquartile range Q1 = 15; Q3 = 65. The diagnosis in five FNAB cases was benign (one normal liver; four non-neoplastic acute or chronic inflammation), one was classified as atypical/suspicious/indeterminate, 52 were malignant and seven samples were insufficient for diagnosis. Table 1 summarises the diagnostic categories outlined.

The majority of lesions were in the abdomen (54/65; 83.1%), followed by pelvis (4/65; 6.1%), chest (2/65; 3.1%), neck (2/65; 3.1%) and miscellaneous sites (3/65; 4.6%) (Table 2). The kidney (20/65; 30.7%) was the commonest organ biopsied or site of primary tumour.

**TABLE 2:** Anatomical location.

Site	Organ	Number
Abdomen (*n* = 54; 83.1%)	Kidney	20
	Retroperitoneum	17
	Liver	10
	Mesentery	5
	Other	2
	Presacral	2
Pelvis (*n* = 4; 6.1%)	Ovary	1
	Peritoneum	1
Chest (*n* = 2; 6.1%)	Anterior mediastinum	1
	Posterior mediastinum	1
	Breast	1
	Paraspinal mass	1
Neck (*n* = 2; 3.1%)	Thyroid gland	1
	Parotid gland	1
Upper limb (*n* = 1; 1.5%)	Arm	1

Table 3 illustrates histologically confirmed tumour types, age distribution and average tumour size at the time of sampling. Nephroblastoma (23/65; 35.4%) was the most common diagnosis, followed by neuroblastoma (8/65; 12.3%), hepatoblastoma (8/65; 12.3%), lymphoma (4/65; 6.2%) and other (10/65; 15.4%). Rhabdomyosarcoma (118 × 100 × 146mm) had the largest average size, followed by nephroblastoma (110.12 × 98.2 × 105.83mm) and hepatoblastoma (99.25 × 108.38 × 136.25mm). From the eight cases of neuroblastoma, two were n-myc amplified, one showed n-myc gain and four were negative. Assessment of n-myc gene amplification was performed using the fluorescent in situ hybridization technique (FISH), with four samples each documented as FNAB cell blocks and bone marrow aspirates respectively. Of the four FNAB cellblock samples, three samples yielded sufficient n-myc classification (n-myc negative = 2 cases; n-myc gain = 1) with unsuccessful analysis in one case.

**TABLE 3:** Histologically confirmed malignant tumours.

Tumour diagnosis	Cases		Age range (months)	Average tumour size‡ (AP × TV × CC in millimetres)
	*n*	%		
Nephroblastoma	23	35.4	8–82	110.12 × 98.20 × 105.83
Neuroblastoma	8	12.3	7–124	86.25 × 96.63 × 93.75
Hepatoblastoma	8	12.3	6–48	99.25 × 108.38 × 136.25
Lymphoma	4	6.2	57–204	89.75 × 87 × 91.5
Desmoplastic small round blue cell tumour (DSRCT)	2	3.1	-	94 × 86 × 120
Yolk sac tumour	2	3.1	-	40.5 × 36.5 × 41.5
Rhabdomyosarcoma	1	1.5	-	118 × 100 × 146
Other†	5	7.7	-	-

† , Includes myoepithelial carcinoma, granulosa cell tumour, Kaposiform haemangioendothelioma, renal rhabdoid tumour, angiosarcoma;

‡ , Average of all 3 orthogonal dimensions (AP, anteroposterior; TV, transverse; CC, craniocaudal).

The average turnaround time for a final FNAB result publication on Lab Trak was 4.7 days, whereas the average turnaround time for a formal histology result was 11.5 days. Provisional/verbal FNAB with ROSE results are, however, typically provided at the time of sampling. There was only one case of a minor peri-procedural complication (1.5%), that is, a small haematoma which resolved on manual compression and required no further intervention.

### Diagnostic yield and accuracy

**FIGURE 1:**
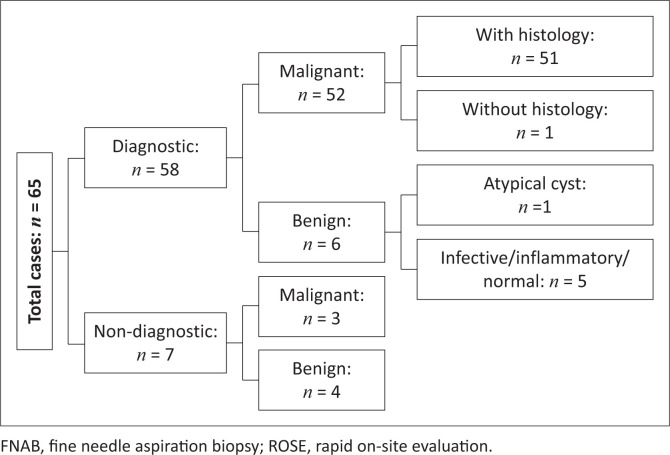
Breakdown of FNAB with ROSE results.

Of the 65 FNAB cases, seven cases (10.7%) were inadequate (no cells or too few cells) for diagnosis to be confirmed with FNAB (Figure 1). Of the remaining 58 cases, 52 (89.7%) were diagnosed as malignancies and 51 (98%) of those had a corresponding histological specimen. The one case that did not have a corresponding histological specimen was diagnosed as nephroblastoma on clinico-radiological grounds and FNAB, however this patient died of sepsis prior to surgical resection, therefore the diagnosis was not confirmed. This case was excluded for the statistical measurement of diagnostic yield and accuracy, as there was no histology available.

The six cases (10.3%) that were not confirmed malignancies by FNAB included one case which was diagnosed as a cyst of indeterminate origin (atypical) with a differential diagnosis of cystic nephroma, cystic partially differentiated nephroblastoma and cystic nephroblastoma. This case was subsequently confirmed to be a cystic nephroblastoma on resection specimen histology. The other five cases were diagnosed as either an inflammatory or infective lesion or normal tissue (Table 1). These cases had no histological follow-up specimen; however, the diagnoses were accepted based on clinico-microbiological-radiological correlation.

Of the seven cases where a diagnosis could not be made with FNAB, one case had no histological follow-up, but was diagnosed as a breast cyst on subsequent ultrasound with complete resolution on short-term follow-up. Three cases turned out to be benign (arteriovenous malformation, fibrous tissue and a benign teratoma). The remaining three cases were confirmed as malignancies on histology, namely cystic nephroblastoma, desmoid fibromatosis and medullary thyroid carcinoma.

Of the 58 cases that had a corresponding histological diagnosis as stated above, 46 cases (79.3 %) had congruent FNAB and histology results for a specific tumour diagnosis, while one case (1.7%) had an incongruent specific diagnosis: the FNAB diagnosis was neuroblastoma whereas the histological diagnosis was nephroblastoma. In 10 of the 58 cases (17.2%), a malignant diagnosis was confirmed via FNAB, but the specific tumour type could not be determined. The one remaining case (1.7%) had a FNAB diagnosis of a paucicellular cyst comprising bland epithelial cells with a cytological differential diagnosis of mesoblastic nephroma, dysplastic kidney or cystic nephroblastoma. The final histological diagnosis was cystic nephroblastoma.

As described above, one case was diagnosed on FNAB as neuroblastoma but after histological review of the resection specimen, the diagnosis was changed to nephroblastoma. This error is thought to have been caused by under sampling, as only the neuroepithelium that was present in the nephroblastoma was aspirated and based on this feature a diagnosis of neuroblastoma was issued. The neuroblastic epithelium was confirmed by immunocytochemical stains on the cell block but the nephroblastoma tissue was not represented on the cytology specimen.

Of the 10 cases diagnosed as malignant on FNAB but where a specific diagnosis could not be determined, non-specific malignant cells were identified in six cases and small round blue cell tumour cells in four cases. The ‘undifferentiated malignant cells’ cases had the following corresponding histological diagnoses: angiosarcoma (*n* = 1); desmoplastic small round blue cell tumour (DSRCT) (*n* = 1), rhabdomyosarcoma (*n* = 1), myoepithelial carcinoma (*n* = 1), Kaposiform haemangioendothelioma (*n* = 1) and non-Hodgkin’s anaplastic large T-cell lymphoma (*n* = 4). For the angiosarcoma, DSRCT and rhabdomyosarcoma cases, the cell block contained no cells and hence no immunocytochemical stains could be performed. For the myoepithelial carcinoma, Kaposiform haemangioendothelioma and lymphoma cases, the cell block contained cells and immunocytochemical stains were performed but a specific diagnosis could not be reached. Of the four cases described as small round blue cell tumour on FNAB, three were histologically confirmed as nephroblastoma, while the remaining one case was confirmed as a neuroblastoma.

The overall diagnostic yield was 98.5%, with a sensitivity of 94.5% and specificity of 100.0%. The diagnostic FNAB accuracy was 95.3%, with a positive predictive value of 100.0% (i.e., there were no false positive diagnoses on FNAB in this series), and a negative predictive value of 75.0%. The low negative predictive value is attributable to the insufficient samples on FNAB. The specific entity diagnostic rate was 72.0%.

### Imaging observations

Fifty-six of the 65 cases (86.2%) were predominantly solid lesions, with six (9.2%) being solid-cystic (complex) lesions and three (4.6%) predominantly cystic lesions. Of note, inadequate samples accounted for 7.1% (four cases out of 56), 16.7% (one case out of six) and 66.7% (two cases out of three) of these categories, respectively.

#### Restricted diffusion

Restricted diffusion on MRI (low apparent diffusion coefficient (ADC) and high diffusion-weighted imaging (DWI) signal at high B values) was reported in 26 cases (40.0%) of which 11 cases were nephroblastoma (42.3%); eight cases hepatoblastoma (30.8%); four cases neuroblastoma (15.4%); and one case each of lymphoma, rhabdomyosarcoma, and DSRCT.

Of note, all 26 cases (100%) that demonstrated restricted diffusion had a cytologically adequate specimen and were confirmed malignant on both cytology and histology, suggesting that highly cellular tumours may be more amenable to a correct FNAB diagnosis as a first line, with both a high diagnostic yield (100%) and accuracy (100%) respectively in this cohort.

#### Nephroblastoma

**FIGURE 2:**
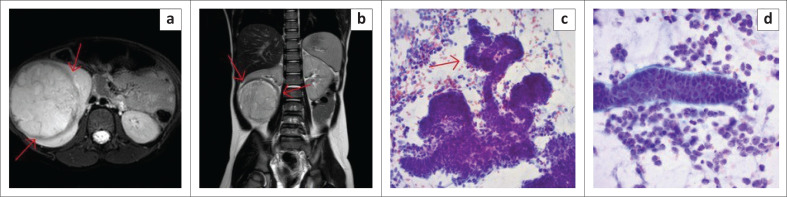
(a) Axial T2W and (b) Coronal T2W images demonstrate a large heterogeneously hyperintense mass with sharp angles (red arrows) on either side of the surrounding normal renal parenchyma described as the ‘claw sign’, in keeping with renal origin. (c) Shows cytological image of blastema cells dispersed with tubule formation (red arrow). The tubules are more rigid and seem to form a tubular structure (Papanicolaou stain 20×). (d) Higher magnification shows a tubule with surrounding basement membrane material (greenish in colour) (Papanicolaou stain 40×).

As mentioned, the most common malignancy was nephroblastoma (23/65; 35.4%) with an average three-dimension orthogonal tumour size of 110.12 × 98.20 × 105.83 mm at the time of imaging (second largest). Of the 24 confirmed primary renal tumours (23 nephroblastoma; one renal rhabdoid tumour), 20 (83.33%) representing the vast majority had an identifiable claw sign on MRI (Figure 2). Of these 20 cases, the primary radiological diagnosis was nephroblastoma in 18 cases (90.0%), with two cystic lesions diagnosed as multilocular cystic nephroma. Of the 18 radiologically diagnosed nephroblastoma cases demonstrating a claw sign, seventeen (94.4%) were subsequently confirmed as nephroblastoma (16 on both FNAB and histology; one on FNAB alone as no resection was performed due to death), with one misdiagnosed case which was subsequently confirmed to be a renal rhabdoid tumour.

**FIGURE 3:**
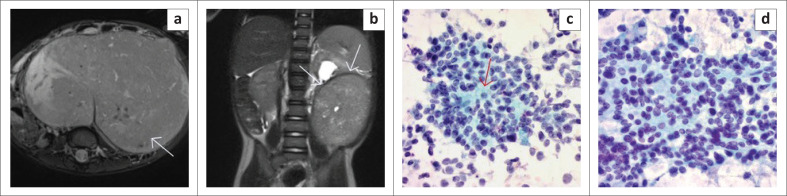
(a) Axial T2W image demonstrated a massive heterogeneous intra-abdominal mass (white arrow), which appeared to arise from the retroperitoneum, and crossed the midline to the contralateral side. Exit foramina were, however, intact. (b) Coronal T2W image of the same patient, demonstrated a left renal claw sign (white arrows), in keeping with renal origin. This claw sign was initially missed which resulted in an incorrect primary radiological diagnosis of neuroblastoma instead of nephroblastoma. (c & d) Cytology shows small blue cells with round-oval nuclei with a pinpoint nucleolus lying associated with a green fibrillar matrix (red arrow) which is neuropil. This was interpreted as neuroblastoma as the only component present on the FNAB (Papanicolaou stain, 40×).

One of the four remaining confirmed renal cases without a claw sign present was the case that was misdiagnosed as neuroblastoma, both radiologically and on FNAB, and for which the resection histology confirmed a diagnosis of nephroblastoma. This misdiagnosis was attributed to the massive nature of the lesion, however when the images were reviewed again, a claw sign was identified on the T2W coronal sequence (Figure 3).

#### Neuroblastoma and hepatoblastoma

**FIGURE 4:**
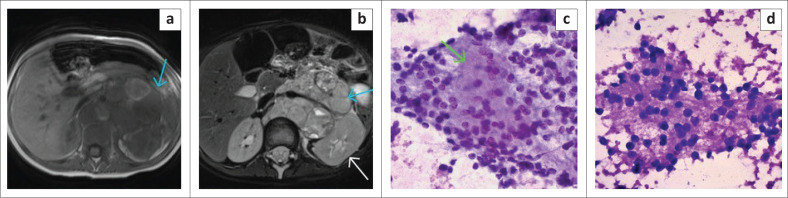
(a) T1W and (b) T2W axial slices demonstrate a large left retroperitoneal mass (blue arrows) with T1W heterogeneously hypointense signal, and areas of increased signal likely representing subacute to chronic haemorrhage; T2W heterogeneously hyperintense signal. The mass anteriorly crosses the midline, displacing and encasing vessels and posterolaterally abuts and displaces the left kidney (white arrow). No claw sign is present. Findings are consistent with a neuroblastoma. (c) Shows a Papanicolaou stain confirming a neuroblastoma with neuroblasts lying pink fibrillary stroma (neuropil) (green arrow) (20×). (d) Shows a Giemsa stain with metachromatic, fibrillary neuropil with small blue neuroblasts (40×).

**FIGURE 5:**
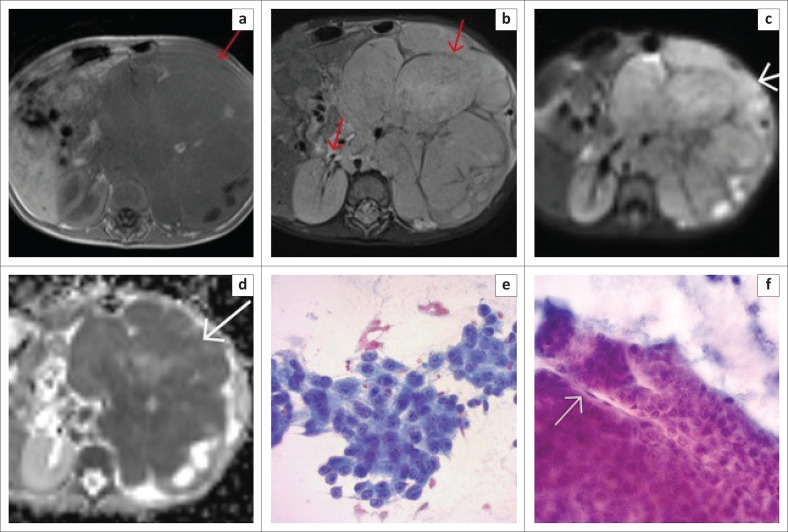
(a) T1W and (b) T2W axial images demonstrate a large lobulated left sided heterogeneous intra-abdominal mass (red arrow heads), T1W hypointense, T2W moderately hyperintense with restricted diffusion (high signal on [c] diffusion-weighted imaging with corresponding low signal on [d] apparent diffusion coefficient - white arrow head). The mass encases and displaces vessels (red arrow), crossing the midline to the contralateral side. No infiltration of neural foramina on limited axial slices. (e) Cytology image showing small primitive hepatocytes in a small, cohesive, tissue fragment. The cells are small with minimal cytoplasm, round nuclei with nucleoli (Papanicolaou stain, 40×). (f) A larger tissue fragment showing hepatoblasts growing in sheets and semi-trabeculae. In one area sinusoidal cells line the edge of the trabeculae (sinusoidal rapping) (white arrow) (Papanicolaou stain, 40×).

**FIGURE 6:**
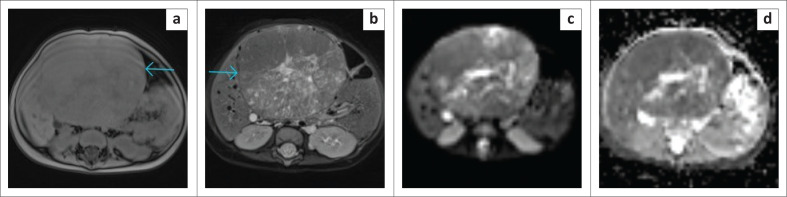
(a & b)T1W and T2W axial images illustrate a large heterogenous T1W hypointense, T2W hyperintense round hepatic mass (blue arrows) replacing the left liver lobe. The mass demonstrates areas of mild restricted diffusion on DWI and ADC sequences (c & d).

The second most common tumours were neuroblastoma (Figure 4 and Figure 5) and hepatoblastoma (Figure 6) with eight cases each (12.3%).

#### Pseudo tumour lesions

**FIGURE 7:**
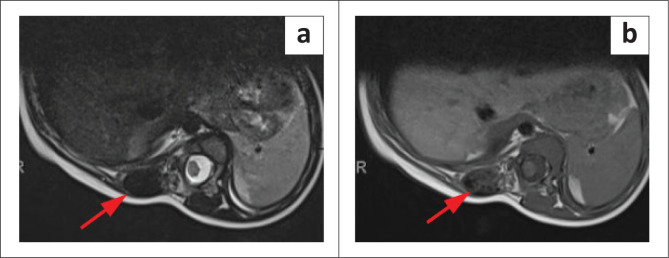
T1W and T2W axial images illustrated a right paraspinal muscle lesion (red arrows), T1W markedly hypointense, and T2W iso to hypointense suspected radiologically to be a mass, subsequently confirmed as benign fibro-connective tissue.

**FIGURE 8:**
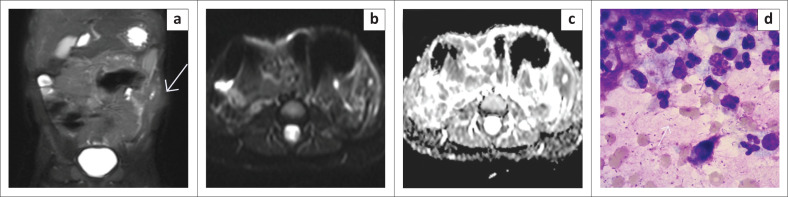
(a) MRI images: T2W coronal image illustrated a heterogenous left flank abdominal wall lesion (white arrow), with a central area of hyperintense signal. The lesion demonstrated restricted diffusion on (b) diffusion-weighted imaging and (d) apparent diffusion coefficient. This lesion, initially suspected to be a mass, was subsequently confirmed to be an abscess on fine needle aspiration biopsy, with an aspirate of pus. (d) Shows a Giemsa stain showing acute and chronic inflammatory cells with numerous bacterial cocci in the background (white arrow) (Giemsa stain, 40×).

Two pseudo tumour lesions were identified, both with an initial radiological diagnosis of rhabdomyosarcoma, which were subsequently confirmed as benign fibro-connective tissue and an abscess on FNAB, respectively (Figure 7 and Figure 8).

## Discussion

In this study, FNAB performed under ultrasound-guidance was quick and relatively easy with only one minor complication observed. The procedure is mostly performed post MRI and CT scan imaging under the same anaesthesia/sedation which in our experience results in increased operational efficiency. The overall diagnostic FNAB yield was 98.0%, with a 72.0% diagnostic accuracy for a specific lesion diagnosis (overall diagnostic accuracy 95.3%) which is comparable to similar other studies.^14,15,16,17,18^ A treatment plan could be initiated in most cases based on a specific diagnosis confirmed via FNAB.

In a resource-limited setting, with a high pressure on theatre facilities, image-guided FNAB with ROSE is a valuable and accurate diagnostic modality as a first line investigative tool. Though CNB is viewed as diagnostically superior and able to provide more tissue for ancillary investigations, FNAB offers a viable alternative with fewer side effects and less risk for tumour spillage^19,20^. Additionally, not all lesions are malignant or lend themselves to CNB, either due to their location and/or vascularity, size, or a poor patient health status. Surgical biopsy performed in theatre is available, but in our setting, there is a trade-off with either a prolonged waiting time for surgery on an emergency theatre list (often days) or postponing the surgical management of another patient already on a routine surgical list to make space for the new patient with a suspected tumour. Hence image-guided biopsies in the radiology department are preferable for expedited diagnosis and initiation of management.

The International Society of Paediatric Oncology (SIOP) treatment protocol for renal tumours does not require a tissue diagnosis for initiation of pre-operative chemotherapy.^21^ We found that performing a FNAB at the time of diagnostic imaging was of value to the paediatric oncology team as it confirmed malignancy in all 22 solid renal lesions, with 21 of the 22 (95.5%) lesions diagnosed correctly. One renal rhabdoid tumour was diagnosed on FNAB while radiologically being suspected as a nephroblastoma. This early correct diagnosis allowed the paediatric oncology team to schedule additional staging investigations required for this malignancy and assisted in providing more accurate information regarding prognosis and the treatment plan, which could be shared with the patient’s parents. In addition, the imaging claw sign is sensitive in accurate identification of an intra-renal mass, however this has a low specificity, particularly for the larger tumour sizes at presentation seen in developing countries.^22^

This study, however, found that multicystic renal lesions do not lend themselves to FNAB, as sampling is inadequate. Two such lesions were confirmed as cystic partially differentiated nephroblastoma and cystic nephroblastoma on histopathology. We suspect that the yield of CNB would not be significantly better in this scenario.

In the age of personalised medicine, FNAB is not always viewed as adequate for a complete diagnosis given a large number of available molecular diagnostics including genetic tests, biomarker tests, and companion diagnostics.^23^ However, in a resource-limited setting, the use of large numbers of tumour biomarkers is not available. Preparation of FNAB cell blocks allows the identification of some biomarkers, making the use of FNAB even more compelling and a very viable alternative. Neuroblastomas are a good example of such a situation. Seven cases were diagnosed correctly with one case being misdiagnosed as a neuroblastoma on FNAB and which turned out to be a nephroblastoma on resection histology. As mentioned, this misdiagnosis was due to under sampling of the tumour with only the neuroepithelial component being sampled. This case was further complicated with a conflicting radiological differential diagnosis and some uncertainty whether it primarily arose in the kidney or affected it secondarily. The authors propose that if there is conflicting radiological-pathological diagnosis that a formal biopsy be performed for a definitive diagnosis. The weakness of FNAB is that it is unable to identify a specific subtype diagnosis and perform the mitotic-karyorrhectic index that is needed for neuroblastoma histological subclassification. However, as most of our neuroblastoma cases are metastatic at presentation often with more than one image-defined risk factor, the FNAB weakness can be mitigated as an International Neuroblastoma Pathology Classification (the Shimada system) is not absolutely required for diagnosis in such cases.^24,25,26^ FNAB yielded valuable information about the n-myc status in three of our cases.

Of the eight hepatoblastomas in this cohort, FNAB managed to diagnose all correctly. Two cases were children less than six months old, three were between six months and three years and the remaining three cases were older than three years. Histology is usually needed prior to commencement of chemotherapy in children younger than six months, older than three years or if a normal serum alpha fetoprotein (AFP) is present.^27,28,29^ As the majority of our hepatoblastoma cases occurred in these age groups (5/8; 62.5%), FNAB assisted in providing a rapid diagnosis after which treatment could be initiated.

Soft tissue sarcomas can be diagnosed on FNAB; however, this may be challenging and the reasons are threefold: (1) reactive cellular / pseudo malignant changes in aspirated surrounding tissues; (2) poor yield or technically inferior smears and more commonly (3) misinterpretation of the aspirated cells due to numerous subtypes and morphological heterogeneity.^30^ One of the DSRCT cases was diagnosed accurately including the Ewing sarcoma breakpoint (EWSR) translocation. In the second case there was no material on the cell block for ancillary investigations. This also held true for the cases of angiosarcoma and rhabdomyosarcoma. In the cases of myoepithelial carcinoma and anaplastic large T-cell lymphoma, the immunostains could not confirm the true nature of these cells and a diagnosis of Kaposiform haemangioendothelioma is deemed extremely difficult as it is a rare tumour.

Key radiological descriptors of tumours include the presence of restricted diffusion, which suggests highly cellular lesions or cellular swelling.^31^ All cases which demonstrated restricted diffusion on imaging, had adequate samples and appropriate diagnoses on FNAB with ROSE, suggesting that high cellularity increases diagnostic yield.

Although FNAB is not the preferred modality of diagnosis in many centres, particularly where there is a need to determine multiple tumour biomarkers; it does, however, offer a viable alternative to other modes of biopsy and is an excellent companion to a CNB. Our cohort shows that FNAB can provide a diagnosis that is adequate for initiation of therapy in many cases. As ancillary techniques evolve and become easier and more affordable, we are certain that FNAB with cell block material will be able to play a larger and more important role in tumour diagnosis and management.

### Study strengths and limitations

The multidisciplinary involvement of pathology, radiology and oncology divisions in this study will potentially result in optimised practices and ultimately improved patient experience and diagnosis. In addition, the retrospective nature of the study reduces the risk of interpreter bias, however on the same note the retrospective nature may not be representative due to the possibility of missed / lost data.

### Future recommendations

An important next step would be to further evaluate the role of imaging findings, in particular the presence of restricted diffusion to guide patient selection for paediatric imaged-guided FNAB with ROSE. It would also be important to explore the impact of the use of FNAB with ROSE on clinical outcomes.

## Conclusion

Paediatric imaged-guided FNAB with ROSE has a relatively high diagnostic yield, sensitivity, specificity, positive predictive value and accuracy in the diagnosis of suspected malignancy. It offers a fast, viable and safe alternative diagnostic modality to diagnose such tumours, particularly when correlated with imaging findings. The relatively low negative predictive value in this study was attributed to a number of insufficient samples, particularly from cystic and benign lesions.
